# Supplementary material for: Downregulation of Trpv4 and Klf2 in brain microvessels is associated with the progression of neurovascular dysfunction and cognitive impairment in a model of heart failure with preserved ejection fraction
Source: J Cereb Blood Flow Metab. 2026 Jun 12:0271678X261462714. Online ahead of print. doi: 10.1177/0271678X261462714 (PMC13380648; doi:10.1177/0271678X261462714)
Supplement: sj-pdf-1-jcb-10.1177_0271678X261462714 – Supplemental material for Downregulation of Trpv4 and Klf2 in brain microvessels is associated with the progression of neurovascular dysfunction and cognitive impairment in a model of heart failure with preserved ejection fraction [file sj-pdf-1-jcb-10.1177_0271678X261462714.pdf]

Supplementary figures

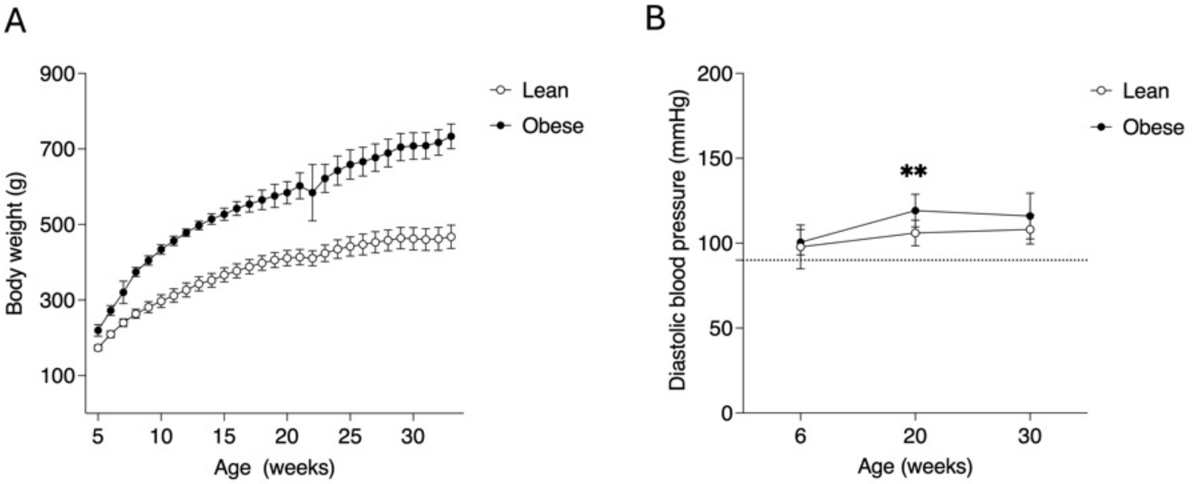

Figure S 1: Progression of body weight and diastolic blood pressure in ZSF1 over time. *(A) Body weight of Lean (open circles) and Obese (filled circles) ZSF1 rats from 5 to 35 weeks of age. (B) Diastolic blood pressure (t=80 mmHg, dashed line (15)) of ZSF1 rats at 6, 20 and 30w: 2-way ANOVA with Sidak's multiple comparisons test:* $p_{age}<0.0001$, $p_{group}=0.0004$, $p_{age\ x\ group}=0.20$. *n=14/group.*

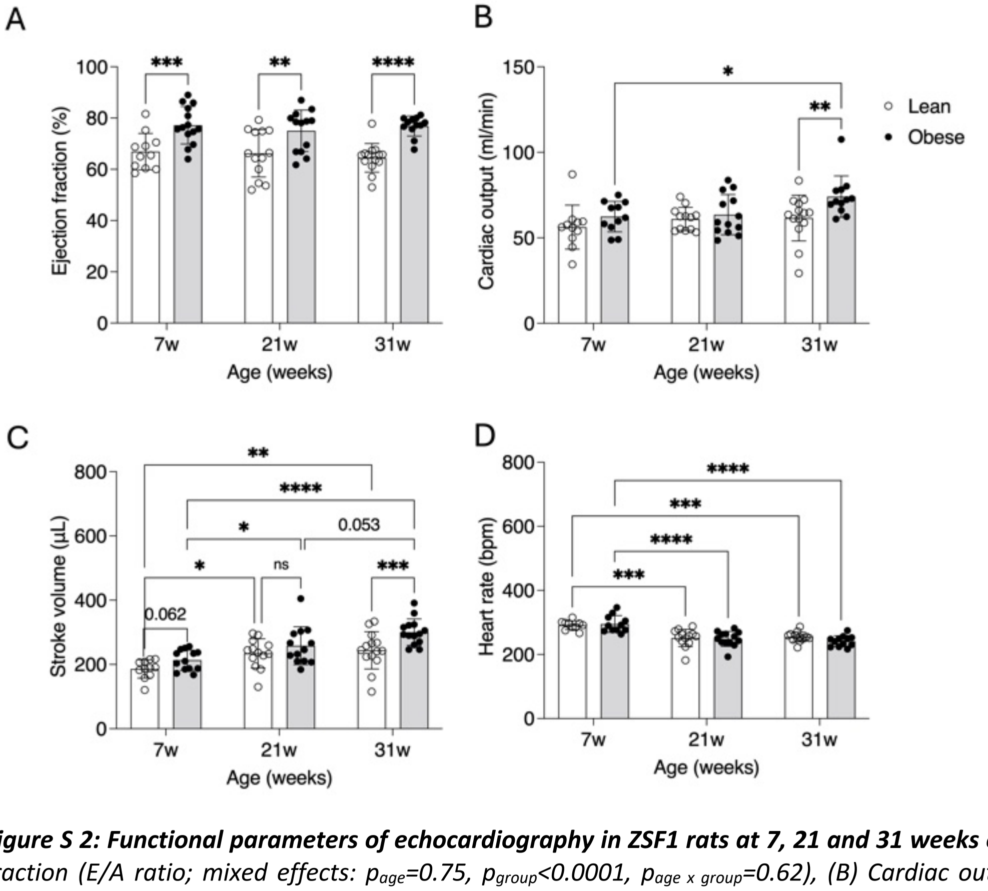

Figure S 2: Functional parameters of echocardiography in ZSF1 rats at 7, 21 and 31 weeks of age*. (A) Ejection fraction (E/A ratio; mixed effects:* $p_{age}=0.75$, $p_{group}<0.0001$, $p_{age\ x\ group}=0.62$*), (B) Cardiac output mixed effects:* $p_{age}=0.0489$, $p_{group}=0.0089$, $p_{age\ x\ group}=0.26$*), (C) Stroke volume (mixed effects:* $p_{age}<0.0001$, $p_{group}=0.0001$, $p_{age\ x\ group}=0.23$*), (D) Heart rate (mixed effects:* $p_{age}<0.0001$, $p_{group}=0.44$, $p_{age\ x\ group}=0.35$*) in Lean (open circles; white bars) and Obese (filled circles; grey bars) ZSF1 rats. Sidak's multiple comparisons test: *p<0.05, **p<0.01, ***p<0.001, ****p<0.0001. n=10-14/group. Bpm = beats per minute.*

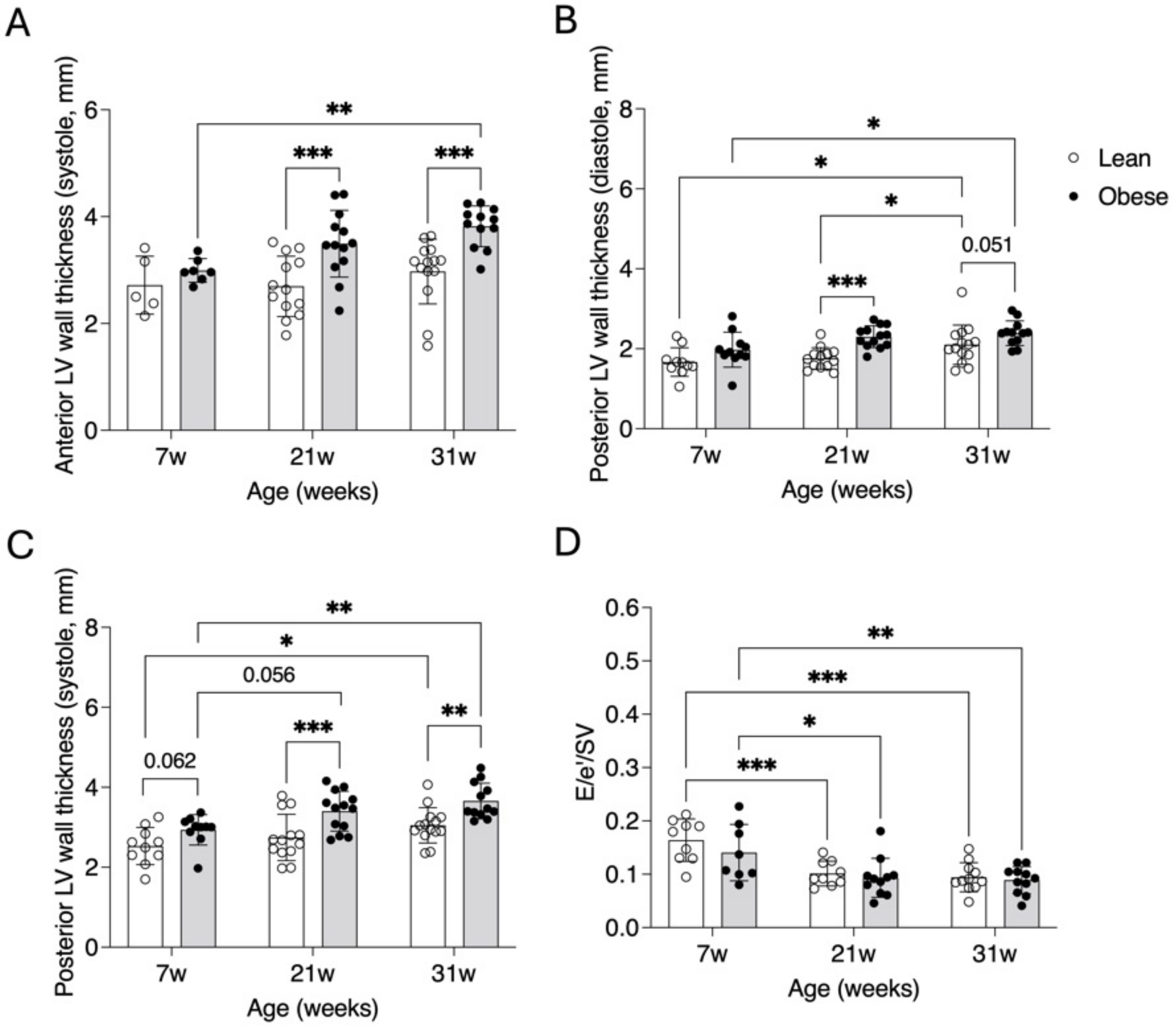

Figure S 3: Left ventricle wall thickness and operant diastolic elastance of ZSF1 rats the age of 7, 21 and 31 weeks. *(A) Anterior left ventricular (LV) wall thickness during systole (mixed effects: p_{age}=0.0133, p_{group}<0.0001, $p_{age\ x\ group}$=0.28), (B) posterior LV wall thickness during diastole (mixed effects: p_{age}=0.0009, p_{group}<0.0001, $p_{age\ x\ group}$=0.38), (C) and systole (mixed effects: p_{age}=0.0009, p_{group}<0.0001, $p_{age\ x\ group}$=0.38), (D) operant diastolic elastance (mixed effects: p_{age}<0.0001, p_{group}=0.18, $p_{age\ x\ group}$=0.69) in Lean (open circles; white bars) and Obese (filled circles; grey bars) ZSF1 rats. Sidak's multiple comparisons test: *p<0.05, **p<0.01, ***p<0.001, ****p<0.0001. n=9-14/group. LV = left ventricle; E = early mitral valve inflow peak velocity; e' = early diastolic mitral annulus peak velocity; SV = stroke volume.*

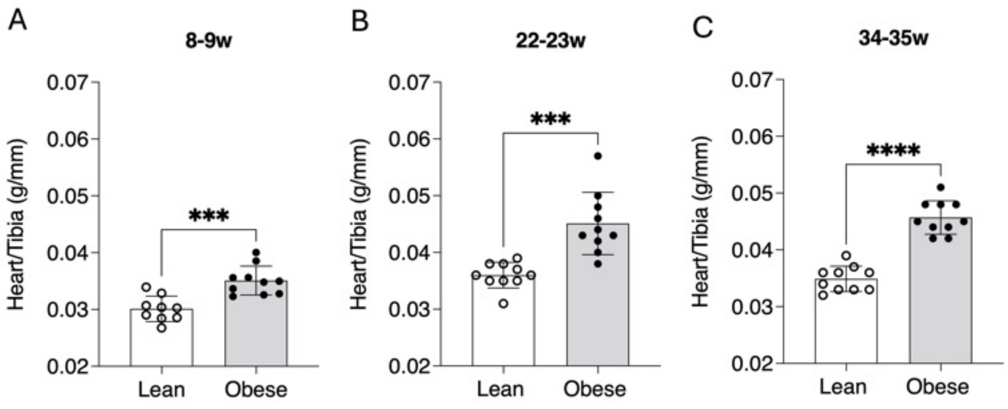

Figure S 4: Cardiac hypertrophy in ZSF1 rats. *Heart weight to tibia length from (A-C) Lean (open circles; white bars) and Obese (filled circles; grey bars) ZSF1 rats. Unpaired t-test: ***p<0.001, ****p<0.0001. n=8-10/group.*

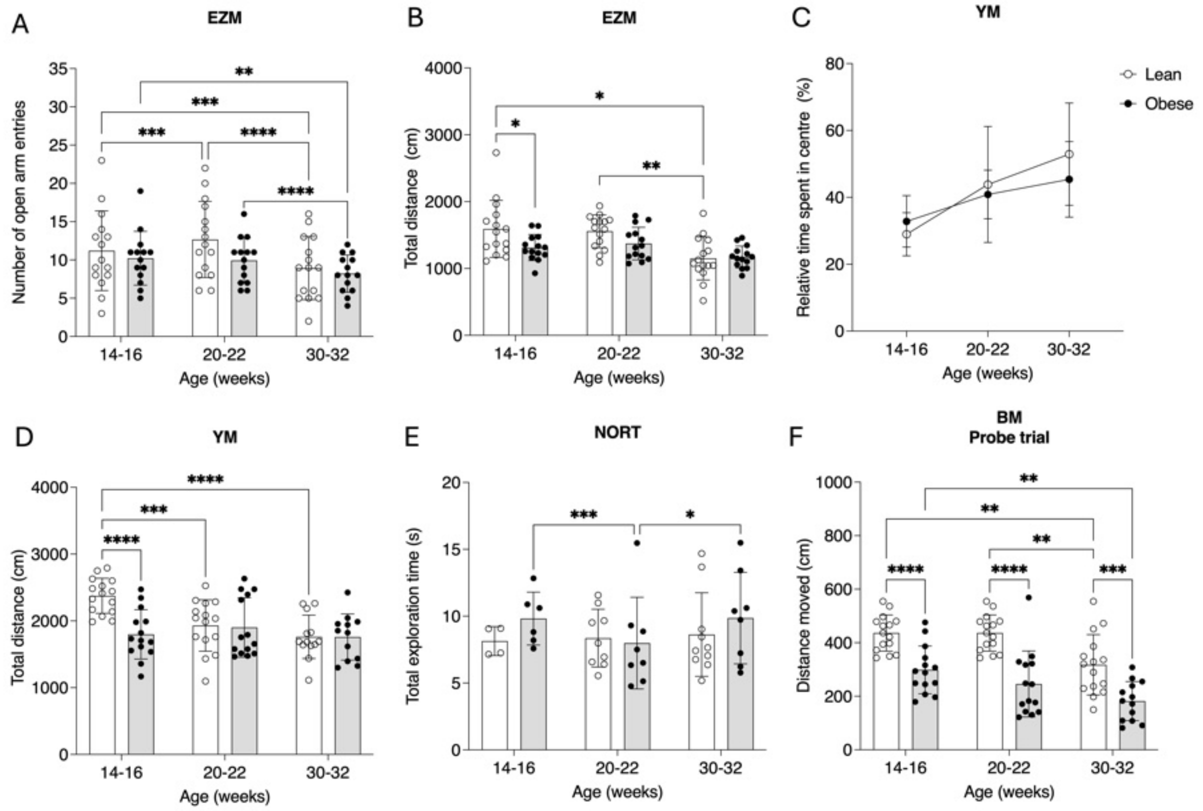

Figure S 5: Cognitive function and locomotor activity of ZSF1 rats at three time points. (A) Number of arm entries of Lean (open circles; white bars) and Obese (filled circles; grey bars) ZSF1 rats during the elevated zero maze (EZM, 2-way ANOVA: $p_{age}<0.0001$, $p_{group}=0.32$, $p_{age \times group}=0.0002$), (B) locomotor activity of during the EZM (2-way ANOVA: $p_{age}=0.0008$, $p_{group}=0.066$, $p_{age \times group}=0.16$), (C) relative time spent in the centre of the Y-maze (YM, mixed effects: $p_{age}<0.0001$, $p_{group}=0.46$, $p_{age \times group}=0.12$), (D) locomotor activity during the YM task (2-way ANOVA: $p_{age}=0.011$, $p_{group}=0.56$, $p_{age \times group}=0.007$), (E) exploration time during the novel object recognition task (NORT, mixed effects: $p_{age}=0.0013$, $p_{group}=0.79$, $p_{age \times group}=0.05$, (F) locomotor activity in the probe trial of the Barnes maze (BM, mixed effects: $p_{age}<0.0001$, $p_{group}<0.0001$, $p_{age \times group}=0.34$). Sidak's multiple comparisons test: *p<0.05, **p<0.01, ***p<0.001, ****p<0.0001. EZM: n=14/15/group, YM: 13-15/group, NORT: n=4-10/group; BM: 13-15/group.

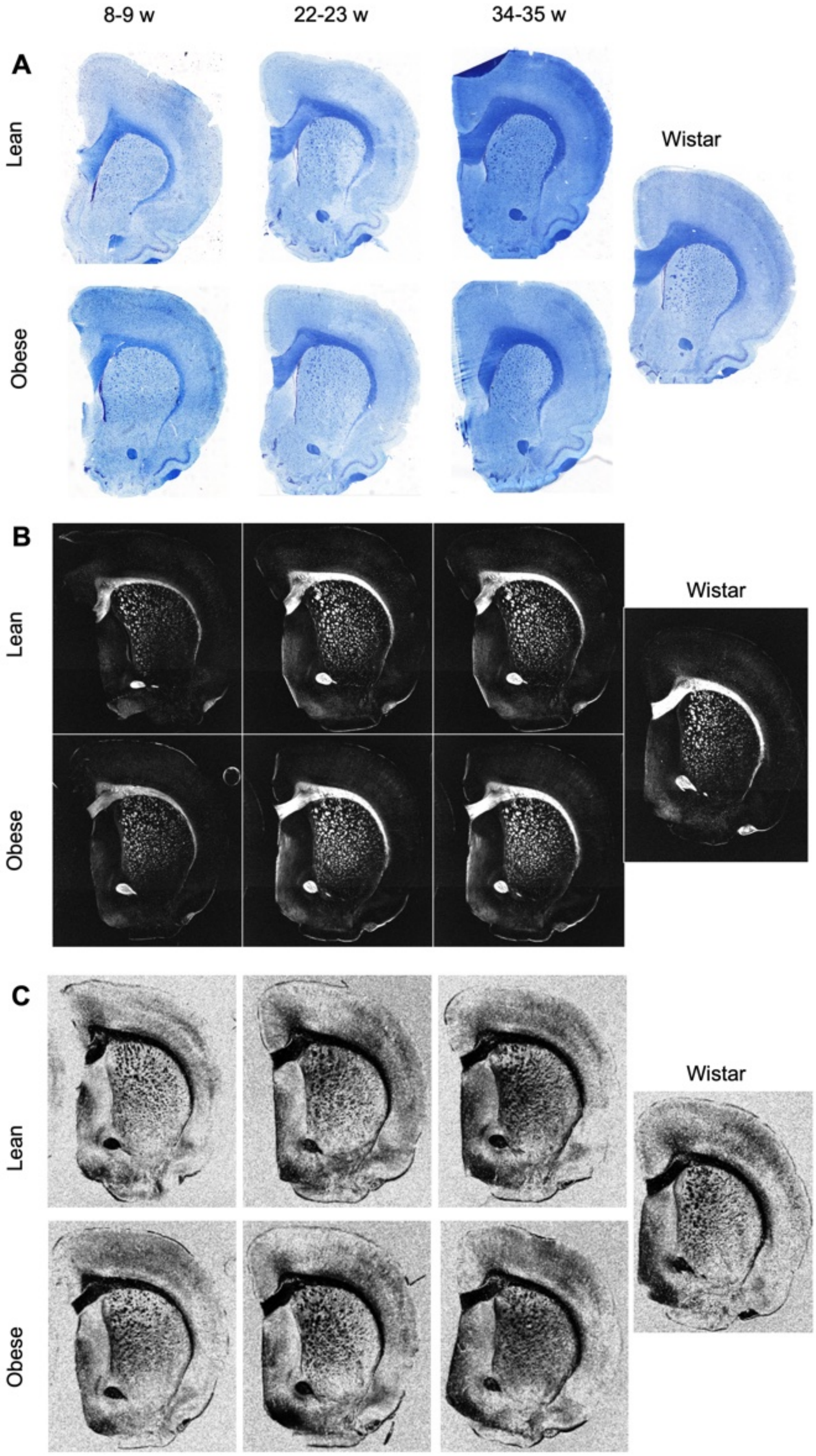

Figure S 6: ***White matter content and integrity in Obese and Lean ZSF1 rats at 8-9 weeks, 22-23 weeks and 34-35 weeks and in Wistar rats***. *(A) Representative images of brain sections stained with Luxol fast blue; Representative images acquired using polarized light imaging depicting (B) retardance and (C) Dispersion*

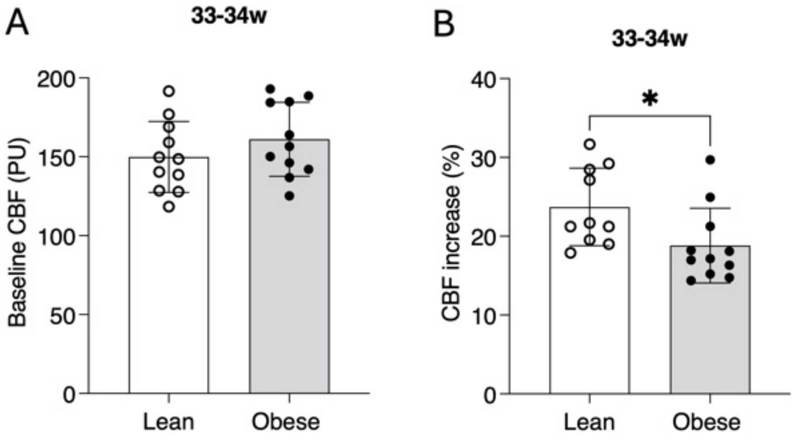

Figure S 7: Cerebral blood flow and neurovascular coupling at 33-34w in ZSF1 and Wistar rats. *In study 1, (A) baseline cerebral blood flow (CBF, unpaired t-test: p=0.26) and (B) neurovascular coupling (unpaired t-test: p=0.03) were measured in Lean (open circles; white bars) and Obese (filled circles; grey bars) ZSF1 rats at the age of 33-34w. Unpaired t-test: *p<0.05. PU = perfusion units. n=10-14/group.*

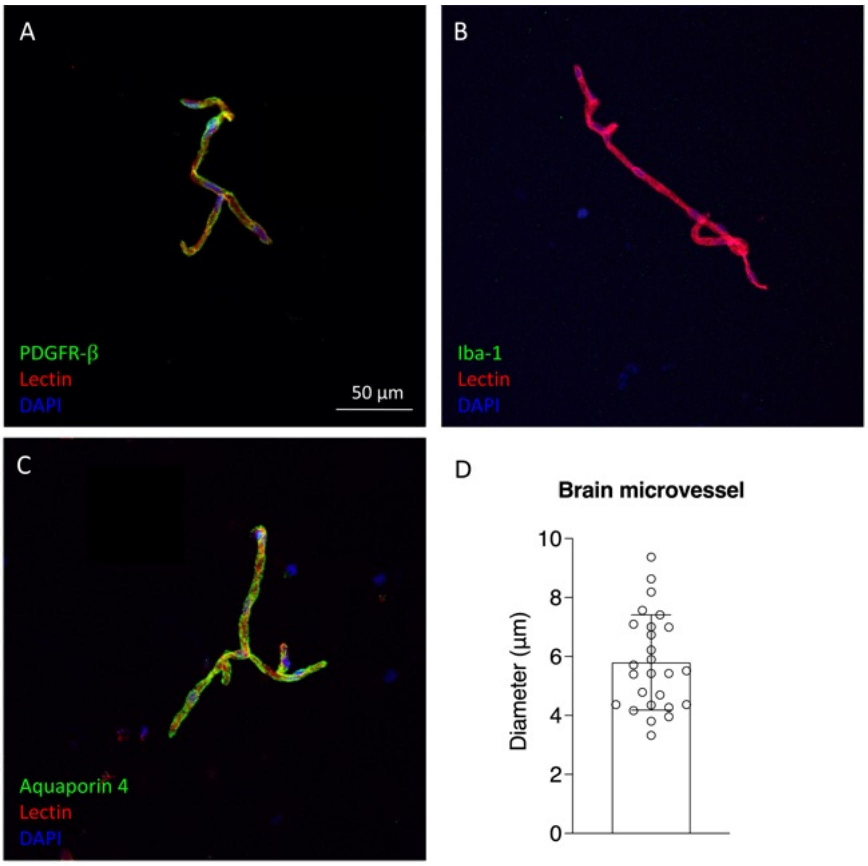

Figure S 8: Characterization of cortical brain microvessels. *Microvessels were fluorescently stained for (A) pericytes using PDGFR-β, (B) microglia using Iba-1 and (C) astrocytic endfeet using aquaporin 4 (green). Blood vessels and cell nuclei were visualized using lectin (red) and DAPI (blue) respectively. (D) Vessel diameter is represented as mean ± SEM (n=27 microvessels). Scale bar = 50 µm.*

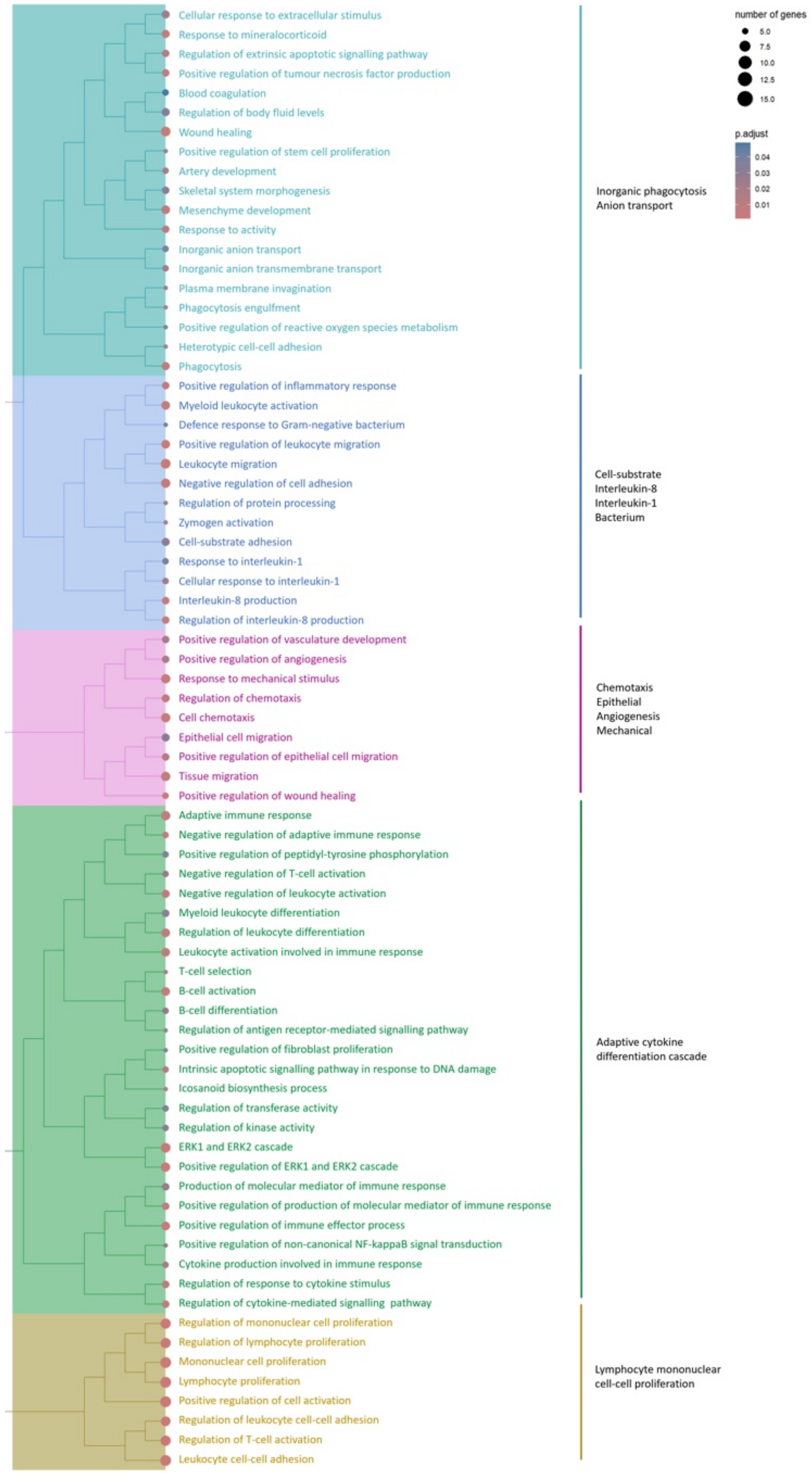

Figure S 9: Overview of functional clusters in Obese vs Lean at 8-9w.

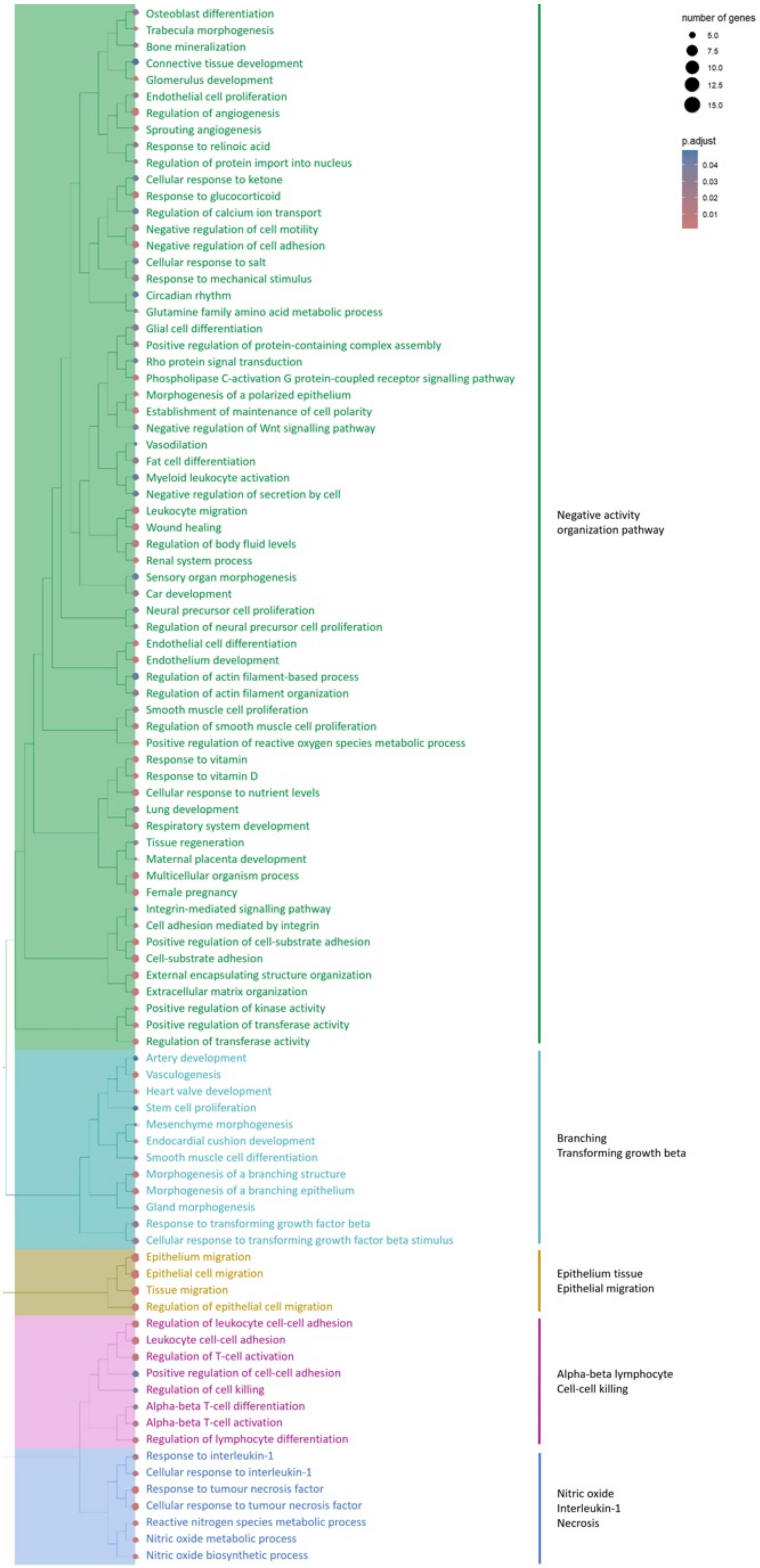

Figure S 10: Overview of functional clusters in Obese vs Lean at 34-35w.

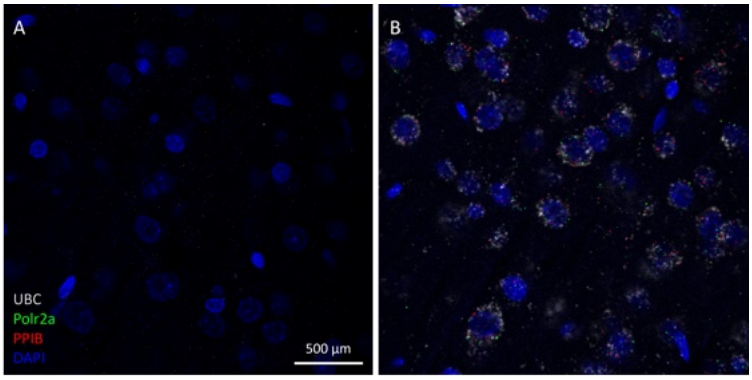

Figure S 11: RNAscope with three-plex control probes. *(A) Negative control; (B) Positive control. Ubiquitin C (UBC) is represented in grey, with medium to high expression levels; DNA-directed RNA polymerase II subunit RPB1 (Polr2a) is represented in green, with low expression levels; Peptidylprolyl isomerase B (PPIB, cyclophilin B) is represented in red, with medium expression levels; Cell nuclei are visualized in blue. Scale bar = 500 µm.*
